# Synchronization of Intracellular Ca^2+^ Release in Multicellular Cardiac Preparations

**DOI:** 10.3389/fphys.2018.00968

**Published:** 2018-07-20

**Authors:** Jessica L. Slabaugh, Lucia Brunello, Mohammad T. Elnakish, Nima Milani-Nejad, Sandor Gyorke, Paul M. L. Janssen

**Affiliations:** ^1^Department of Physiology and Cell Biology, College of Medicine, The Ohio State University, Columbus, OH, United States; ^2^Davis Heart Lung Research Institute, The Ohio State University, Columbus, OH, United States; ^3^Department of Pharmacology and Toxicology, Faculty of Pharmacy, Helwan University, Cairo, Egypt

**Keywords:** trabeculae, calcium, muscle, transient, EC coupling, rat

## Abstract

In myocardial tissue, Ca^2+^ release from the sarcoplasmic reticulum (SR) that occurs via the ryanodine receptor (RyR2) channel complex. Ca^2+^ release through RyR2 can be either stimulated by an action potential (AP) or spontaneous. The latter is often associated with triggered afterdepolarizations, which in turn may lead to sustained arrhythmias. It is believed that some synchronization mechanism exists for afterdepolarizations and APs in neighboring myocytes, possibly a similarly timed recovery of RyR2 from refractoriness, which enables RyR2s to reach the threshold for spontaneous Ca^2+^ release simultaneously. To investigate this synchronization mechanism in absence of genetic factors that predispose arrhythmia, we examined the generation of triggered activity in multicellular cardiac preparations. In myocardial trabeculae from the rat, we demonstrated that in the presence of both isoproterenol and caffeine, neighboring myocytes within the cardiac trabeculae were able to synchronize their diastolic spontaneous SR Ca^2+^ release. Using confocal Ca^2+^ imaging, we could visualize Ca^2+^ waves in the multicellular preparation, while these waves were not always present in every myocyte within the trabeculae, we observed that, over time, the Ca^2+^ waves can synchronize in multiple myocytes. This synchronized activity was sufficiently strong that it could trigger a synchronized, propagated contraction in the whole trabecula encompassing even previously quiescent myocytes. The detection of Ca^2+^ dynamics in individual myocytes in their *in situ* setting at the multicellular level exposed a synchronization mechanism that could induce local triggered activity in the heart in the absence of global Ca^2+^ dysregulation.

## Introduction

The sarcoplasmic reticulum (SR) Ca^2+^ release complex comprises more than a dozen proteins, with the ryanodine receptor (RyR2) as the core channel. The RyR2 has numerous binding sites for regulatory molecules and accessory proteins with the capability to modulate RyR2 function. Ca^2+^ release from the SR is a key determinant of cardiac contractility and both the cytoplasmic as well as the luminal Ca^2+^ concentration can regulate the RyR2. In the SR lumen, in particular, there is a substantial amount of Ca^2+^ buffering, and, according to several studies, approximately 50–90% of the total Ca^2+^ is bound to SR Ca^2+^ binding proteins, such as calsequestrin (CASQ2) (Shannon and Bers, 1997; Shannon et al., 2000). Therefore, there is only a small fraction of free Ca^2+^ in the lumen of the SR (Bers, 2001), and, ultimately, the free Ca^2+^ present in the SR is the driving force for SR Ca^2+^ release (Gyorke and Terentyev, 2008).

As SR Ca^2+^ release is diminished, the open probability of the RyR2 decreases. The temporary, unresponsive state that the RyR2 enters after SR Ca^2+^ release is important to the mechanical refractoriness that is required for effective relaxation and refilling of the ventricles (Biesiadecki et al., 2014). Systolic SR Ca^2+^ release must terminate with a substantial pool of Ca^2+^ still in the SR, and spontaneous Ca^2+^ release should not occur during diastole in order to maintain an efficient control of the SR Ca^2+^ release process (Gyorke, 2009). Although the exact mechanism responsible for Ca^2+^ release termination remains to be elucidated, a significant amount of evidence suggests that changes in luminal Ca^2+^ play a key role in the termination process (Gyorke and Terentyev, 2008). RyR2 modulation by luminal Ca^2+^ has been shown to involve several key proteins associated with the RyR2 in the lumen of the SR, most importantly CASQ2. CASQ2 not only acts as a Ca^2+^ buffer, but it also mediates the responsiveness of the RyR2 channel to luminal Ca^2+^ by serving as a Ca^2+^ sensor (Gyorke et al., 2004; Gyorke and Terentyev, 2008).

When the RyR2-mediated SR Ca^2+^ release is not properly maintained, a variety of cardiac diseases can occur, specifically triggered arrhythmias. When the SR Ca^2+^ content is increased dramatically, the RyR2 becomes much more sensitive to the intracellular cytosolic calcium concentration ([Ca^2+^]_i_), and there is an increase in the open probability. This enhanced RyR2 open probability can result in an increased Ca^2+^ release during systole; however, in diastole, it can cause an inadvertent SR Ca^2+^ leak (Shannon et al., 2002). Also, some acquired or genetic defects found in both RyR2 or its regulatory proteins can lead to a state of “perceived” SR Ca^2+^ overload, where there is a decrease in the threshold for SR Ca^2+^ release that leaves the RyR2 hyperactive (Gyorke and Carnes, 2008). Both of these scenarios can lead to triggered arrhythmias.

Triggered arrhythmias can originate from delayed afterdepolarizations (DADs) and early afterdepolarizations (EADs), and they are initiated by variations in the membrane potential (Venetucci et al., 2006; Gyorke and Carnes, 2008). DADs are most frequently observed under conditions of SR Ca^2+^ overload, where the high SR Ca^2+^ content can cause spontaneous Ca^2+^ release after the termination of the action potential (AP). The Ca^2+^ release from the SR increases the Ca^2+^ concentration in the cytoplasm, allowing for the activation of the Na^+^/Ca^2+^ exchange (NCX), which pumps the Ca^2+^ that leaked from the SR out of the myocyte. Since the NCX ratio is three Na^+^ ions to one Ca^2+^ ion, the membrane potential becomes more positive and can produce transient depolarizations causing DADs (Lederer and Tsien, 1976; Schlotthauer et al., 1998).

For the activation of extra-systolic contractions (ESCs) in a single myocyte, the transient current must be large enough to reach the threshold for AP generation. However, this situation is much more complicated at the whole heart level. Since myocytes are electrically coupled by gap junctions, any depolarization that arises in one myocyte would most likely be dampened due to dissipation of the current in the neighboring myocytes; therefore, the likelihood of triggering an ectopic AP is low. It has been proposed that a DAD would have to arise in approximately 1000 myocytes for an ectopic beat to take place (Winslow et al., 1993). Several other studies have calculated that a in a three-dimensional setting, a minimum of 700,000 to 800,000 myocytes are required (Plotnikov et al., 2007; Xie et al., 2010). Thus, it is believed that some synchronization mechanism exists that has the ability to synchronize DADs and APs in neighboring myocytes, possibly by a similarly timed recovery of the RyR2 from its refractory state, which enables the RyR2 to reach the threshold for spontaneous Ca^2+^ release simultaneously. We recently showed that such a mechanism can occur in genetically mutated murine myocardium (Brunello et al., 2013), but it is unknown if this is a potentially general mechanism that can occur in the absence of genetic mutations. We now hypothesize that this synchronization mechanism that leads to arrhythmias can also occur in multicellular rat myocardial preparations and can occur in the absence of underlying genetic factors that predispose to arrhythmias. Hence, we investigated the properties of non-externally stimulated Ca^2+^ waves and force generation in multicellular trabeculae isolated from healthy rat myocardium.

## Materials and Methods

All protocols were approved by the institutional laboratory animal care and use committee.

### Trabeculae Isolation

Male, Brown Norway rats (approximately 200–300 g) were anesthetized using an intraperitoneal injection of euthasol (392 μg pentobarbital and 40 μg phenytoin). Heparin (1000 U) was injected at the apex of the left ventricle after the chest had been opened by bilateral thoracotomy. The heart was quickly removed and placed in a Krebs–Henseleit (KH) buffer containing (in mM) 137 NaCl, 5 KCl, 1.2 NaH_2_PO_4_, 1.2 MgSO_4_, 20 NaHCO_3_, 0.25 CaCl_2_, and 10 Glucose. 2,3-Butanedione monoxime (BDM, 20 mM) was added to the KH buffer to minimize the cutting injury (Mulieri et al., 1989), as exposure to BDM for a short period of time has been shown to be reversible (Zimmermann et al., 1996; Janssen et al., 2002). Hearts were cannulated via the ascending aorta and perfused using KH buffer with the addition of BDM. This buffer was kept in equilibrium with 95% O_2_ and 5% CO_2_ and constant pH of 7.4. The right ventricle was opened, and thin, uniform, non-branched trabeculae were extracted and mounted as previously described (Slabaugh et al., 2012). The muscle was bathed in fresh, oxygenated KH solution without BDM and containing 1.5 mM Ca^2+^, and stimulated at a frequency of 1 Hz at room temperature until contractile parameters had stabilized.

### Force Measurements

The trabeculae were stretched to a length where further stretching raised diastolic and systolic force proportionally (Monasky et al., 2010). It has been shown that this muscle length corresponds with a sarcomere length of around 2.2–2.3 μm (Allen and Kentish, 1985; Rodriguez et al., 1992), reflecting a typical end-diastolic sarcomere length. The cross-sectional area of the trabeculae was utilized to normalize the developed force to allow for comparison between muscles of different diameters. Muscles with a width of 100–150 μm were specifically chosen to avoid core hypoxia that has been shown to be present in muscles with a width greater than 150 μm (Raman et al., 2006). After the contractile parameters of the muscle had stabilized at 1 Hz (∼20 min), 0.5 mM caffeine, and 100 nM isoproterenol were added to the perfusion solution. Twitch contractions were monitored and recorded throughout the experimental procedures.

### Intracellular Ca^2+^ Measurements

Isolated rat trabeculae were loaded with 40 μM Rhod-2 AM, which was dissolved in 2% pluronic (20% w/v) in 1 mL KH buffer with the addition of TPEN (4.3 mg/L) and cremophor (5 mg/L), in a bath for 45 min at room temperature (Brunello et al., 2013). The electrical stimulation was turned on at 1 Hz during the last 5 min of incubation. After this loading period, the muscle was slacked and perfused with a KH solution containing 5 mM BDM and 20 μM blebbistatin to stop cell contraction and allow for Ca^2+^ imaging (Kovacs et al., 2004). In order to optimize the resolution and minimize motion-induced artifacts, a modified glass capillary tube connected to a micromanipulator was utilized to gently push the trabecula against the coverslip. All data were collected using a 60× water objective, and the line-scan images were acquired at the rate of 8 μs/pixel. The fluorescence emitted was expressed as Δ*F*/*F*_0_ [(*F*-*F*_0_)/*F*_0_], where *F* is the fluorescence at time *t* and *F*_0_ represents the background signal.

**FIGURE 1:**
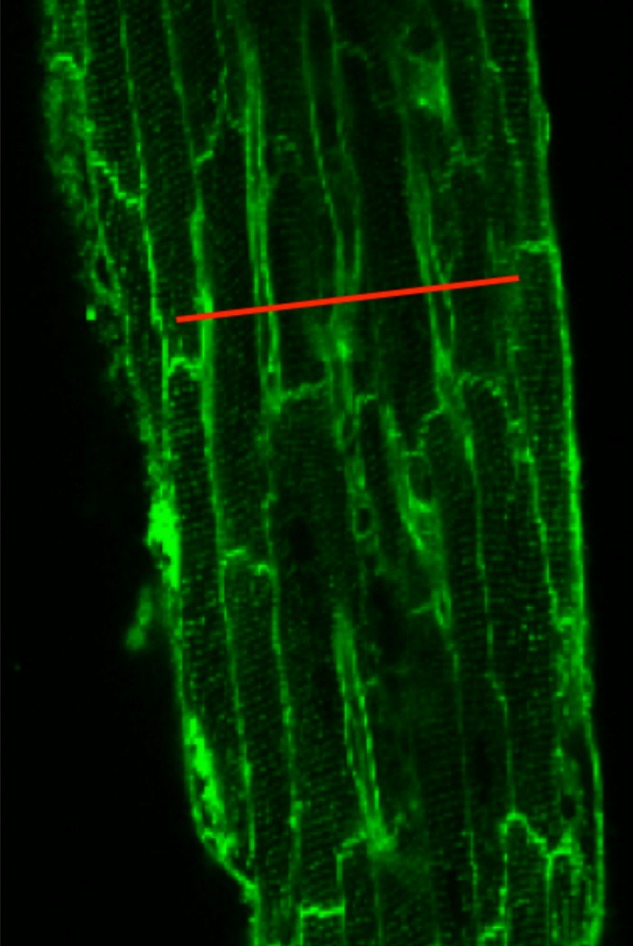
Di-4-ANEPPS loaded trabeculae show the myocyte borders in a rat trabecula in field-scan mode on a confocal microscope. The red line is drawn to illustrate where a typical line-scan would be conducted in experiments. Given the spatial and temporal resolution used, the line-scan typically covered five to seven adjacent myocytes.

In separate experiments, we performed 2D scanning of the entire muscle at the same depth the Ca^2+^ transients are typically measured. In order to confirm that the line-scan represented five to seven myocytes in the center part of the preparation, trabeculae were loaded with di-4-ANEPPS to identify myocytes borders, as shown in **Figure 1**.

### Data Analysis

Data were collected and analyzed using LabView, ImageJ, and Origin software. All data are presented as mean ± SEM, unless otherwise stated. All experiments were performed using protocols approved by The Ohio State University Laboratory Animal Care and Use Committee.

## Results

### Effect of Isoproterenol and Caffeine on Contraction

**FIGURE 2:**
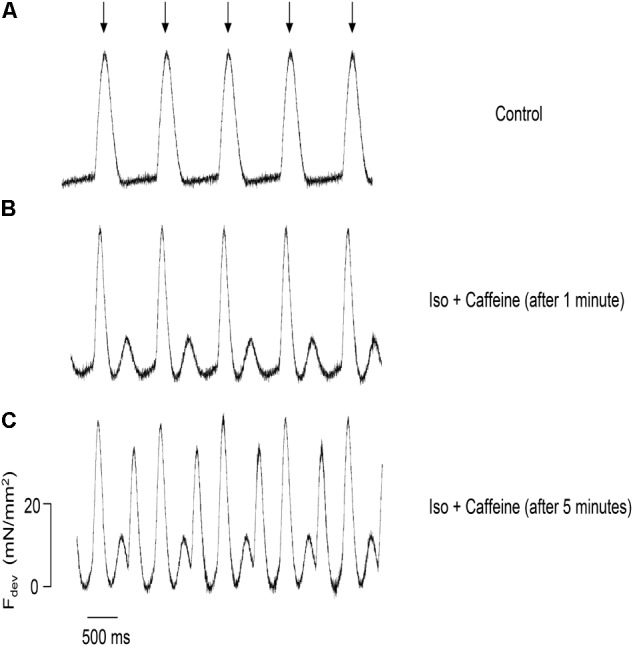
Representative force tracings of a trabecula paced at 1 Hz, with the stimulation indicated by the arrows, applicable to all panels. **(A)** The control trabecula shows no ESCs following relaxation. **(B)** ESCs are observed with the addition of 0.5 mM caffeine and 100 nM isoproterenol. **(C)** Same trabecula after 5 min: the incidence and amplitude of ESCs increase, reaching a maximum of three contractions per cycle.

To investigate the effect of both isoproterenol and caffeine on the incidence of ESCs in trabeculae, we monitored the contractile activity in these muscles while electrically pacing at 1 Hz. As expected, the control, untreated muscle showed no signs of ESCs following relaxation (**Figure 2A**); however, after 1 min of perfusion with 100 nM isoproterenol and 0.5 mM caffeine the trabeculae displayed ESCs between successive twitches (**Figure 2B**). Upon application of both isoproterenol and caffeine, the developed force remained unchanged in the muscles as compared with control, most likely due to the diastolic Ca^2+^ release diminishing the SR Ca^2+^ store. Thus, ESCs in the trabeculae occurred in a highly synchronized manner leading to a spontaneous contraction with characteristics similar to those of the stimulated twitch in both amplitude and relaxation timing (Biesiadecki et al., 2014). Over time, the incidence of ESCs increased, reaching a maximum of three ESCs per cycle, with typically two ESCs occurring per cycle (**Figure 2C**). We found that not only does the frequency of ESCs increase with time but that the amplitude is altered as well.

### Amplitude and Latency Period Distribution

**FIGURE 3:**
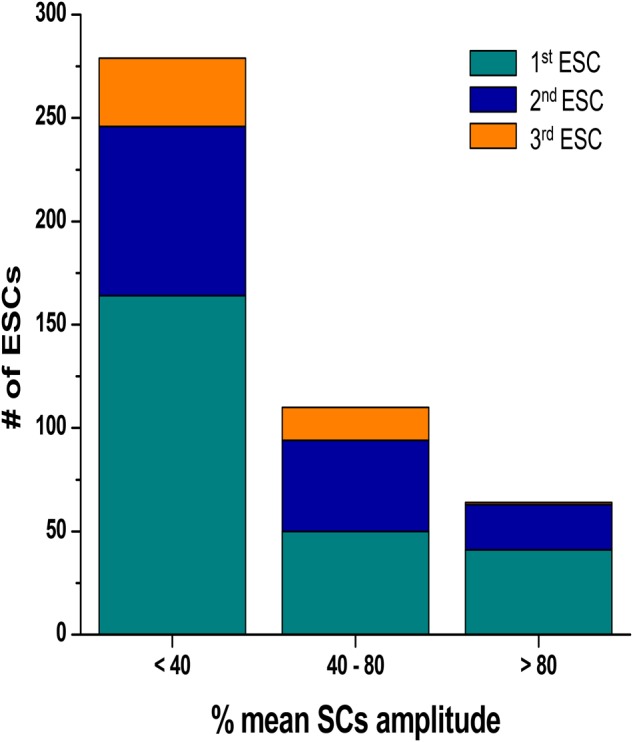
Distribution of the ESCs amplitude as a percentage of the amplitude of the developed force in stimulated contraction. The ESCs were divided into three separate groups according to their temporal order. SC, stimulated contraction.

**FIGURE 4:**
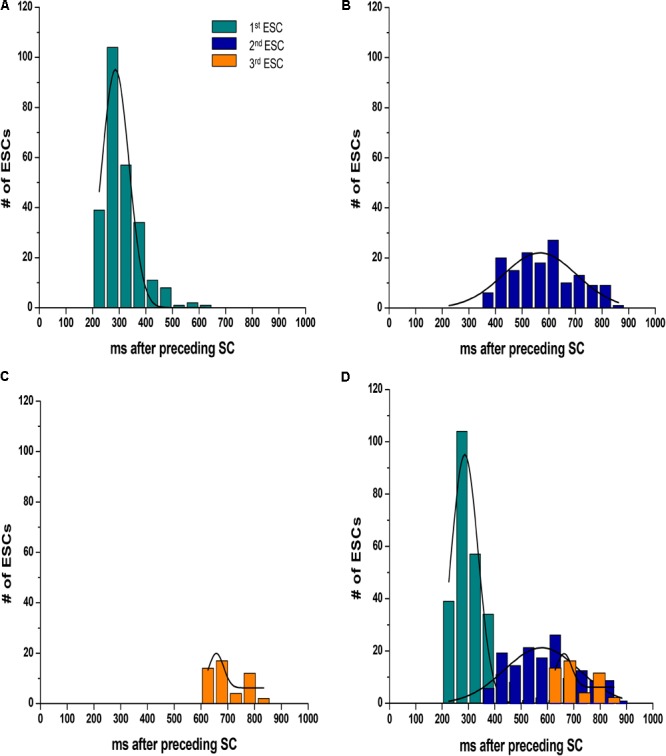
The ESCs are not randomly spread throughout diastole, as they displayed a high probability of occurrence around three specific time points. **(A–C)** Show the temporal distribution of the first, second, and third ESC, occurring at 310 ± 4 ms, 578 ± 10 ms, and 702 ± 10 ms, respectively. The plots are overlaid in **D**. SC, stimulated contraction.

We calculated both the ESC amplitude and the time from the stimulated contraction to the start of the ESC, or latency period from recordings from *n* = 16 rats, 10–20 s of recordings per rat. During each diastolic period, we observed up to three ESCs, and these ESCs were divided into separate groups for analysis. **Figure 3** illustrates the distribution of the amplitude of the ESCs as a percentage of the stimulated contraction’s amplitude. The majority of the ESCs (62%) have an amplitude of less than 40% of the developed force of stimulated contractions. However, 24% of the ESCs showed an amplitude between 40 and 80%, while 14% of ESCs showed and amplitude 80% or higher. Notably, the majority of the ESCs with the highest amplitude occurred within the first ESC group. Although we were not able to simultaneously measure the membrane potential, we hypothesize that many of these high-amplitude ESCs would synchronize and then could result in extra-systolic APs triggered in the trabeculae. In a previously published study by our group (Brunello et al., 2013), performed in parallel to this study, we indeed showed that in a genetic model of disease, some of these ESC’s generated APs. Therefore, these results suggest that the addition of both isoproterenol and caffeine can lead to ESCs in rat myocardium. The ESCs displayed a high probability of occurrence around two or sometimes even three specific time points (**Figure 4**). The latency distribution shows a period of refractoriness after the stimulated twitch, when no ESC occurred, followed by the presence of the first ESC at 310 ± 4 ms (**Figure 4A**). The second ESC occurred at 578 ± 10 ms (**Figure 4B**) while the third ESC appeared at 702 ± 10 ms (**Figure 4C**). Therefore, these results show that the ESCs were highly temporally aligned, which is consistent with a synchronized recruitment of myocytes in the multicellular preparation. The number of waves, and amplitude, varied from muscle-to-muscle, likely due to the various sizes of cross-sectional areas of muscle used, and also increased with time of exposure.

### Effect of Isoproterenol and Caffeine on [Ca^2+^]_i_

**FIGURE 5:**
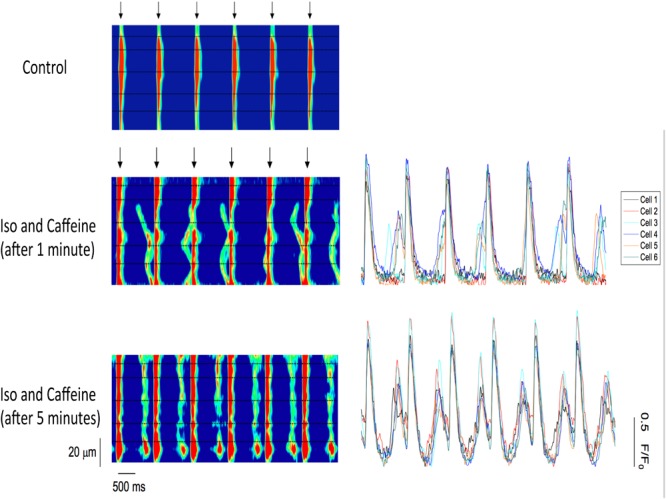
**(Left)** Confocal line-scan images of a cardiac trabecula loaded with the Ca^2+^ dye Rhod-2 and paced at 1 Hz (stimulation indicated by the arrows). The black dotted lines represent myocyte borders, and they were manually drawn based on the deflection of the Ca^2+^ waves. The electrical stimulation triggers SR Ca^2+^ release simultaneously across each of the myocytes in the field of view (control). Following the addition of both 0.5 mM caffeine and 100 nM isoproterenol, the trabecula shows Ca^2+^ waves that occur between stimulated Ca^2+^ release. The Ca^2+^ waves occur only in four out of six cells. However, after 5 min, the Ca^2+^ waves synchronize as the Ca^2+^ release occurs simultaneously across all six myocytes. **(Right)** Confocal line-scan images with the corresponding Ca^2+^ fluorescence plot profiles for each of the six myocytes in the field of view. Top: the Ca^2+^ release is not simultaneous across the myocytes, as the fluorescence peaks occur at different time points and not in all cells. Lower: the fluorescence peaks at the same time in each one of the six cells in the field of view, showing synchronization.

**FIGURE 6:**
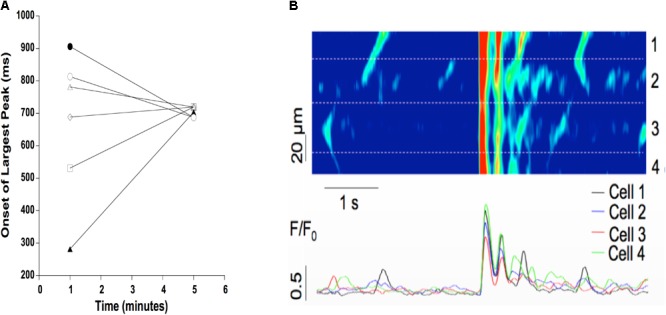
**(A)** Typical example of time of onset of largest ESC at 1 and 5 min after addition of isoproterenol and caffeine. Time of onset is highly variable in six adjacent myocytes after 1 min (spread of onset 100’s of ms), but becomes more synchronized at 5 min (spread of onset of ∼30 ms). **(B)** A non-externally stimulated action potential causes the spread of onset to virtually disappear, i.e., the spread of onset is 0–1 ms between adjacent cells (four in this example) in a triggered event.

To further examine the synchronization of Ca^2+^ release, we loaded cardiac trabeculae with the Ca^2+^ indicator Rhod-2. **Figure 5** shows a line-scan image of Ca^2+^ changes in a single trabecula. As expected, the electrical stimulation triggers SR Ca^2+^ release simultaneously across each of the myocytes in the field of view. Following the addition of isoproterenol and caffeine, the trabecula shows spontaneous Ca^2+^ waves that occur between the stimulated Ca^2+^ release. These Ca^2+^ waves have a lower amplitude than the induced Ca^2+^ transients and, while they occur in several of the cells, they do not occur simultaneously across each of the myocytes. However, after 5 min the Ca^2+^ waves synchronize as the Ca^2+^ release occurs much more uniformly in the myocytes. **Figure 5** also shows the line-scans and corresponding Ca^2+^ fluorescence plot profiles for each of the individual myocytes in the field of view. The fluorescent plot profiles in the top panel demonstrate that each of the diastolic Ca^2+^ release events in the cells behaves differently, as the fluorescence signal from the Ca^2+^ waves peaks throughout the entire diastolic period. As the Ca^2+^ release becomes synchronized, the fluorescence peaks at the same time in each of the myocytes. Consistent with our force experiments, these results indicate that there is a synchronized diastolic Ca^2+^ release that occurs across each of the myocytes in the field of view (**Figure 5**). In **Figure 6A**, we show a typical example of the time of onset of the largest ESC at 1 min, and after 5 min in six adjacent cells. This shows that over time, the adjacent cells seem to synchronize their ESC, i.e., onset of ESC grows closer and closer in all adjacent cells. Still, after 5 min, there is remains some little variation in time of onset (up to 32 ms in this example) in the adjacent cells, indicating synchronization that is not directly due to an AP. In **Figure 6B**, it can be seen that a non-externally stimulated AP causes the adjacent cells (four in this example), to all start the non-externally stimulated event at identical timepoint (0–1 ms variation among the four cells).

**FIGURE 7:**
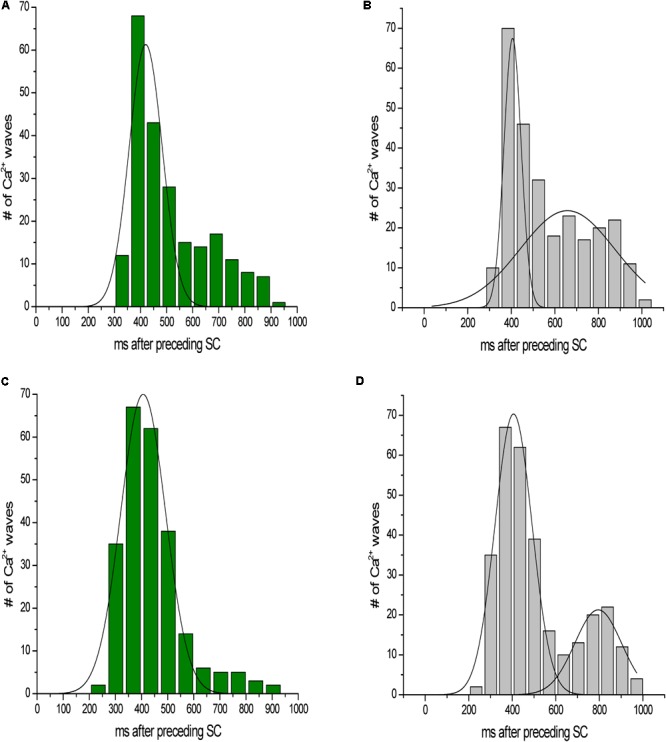
Latency distribution of Ca^2+^ waves. **(A,C)** Latency distribution of the first Ca^2+^ wave at 0.2 and 0.5 mM caffeine, respectively. **(B,D)** Distribution of all waves at 0.2 and 0.5 mM caffeine, respectively.

We next investigated the myocytes response to a lower dose of caffeine (**Figure 7**). At 0.2 mM caffeine, the distribution of the latency of the first Ca^2+^ wave was shifted slightly rightward, compared to the distribution at 0.5 mM caffeine. The distribution of all waves had a similar behavior. In addition, the number of waves per cycle decreased significantly at the 0.2 mM caffeine concentration.

### Role of Systolic Ca^2+^ Release in Synchronization

**FIGURE 8:**
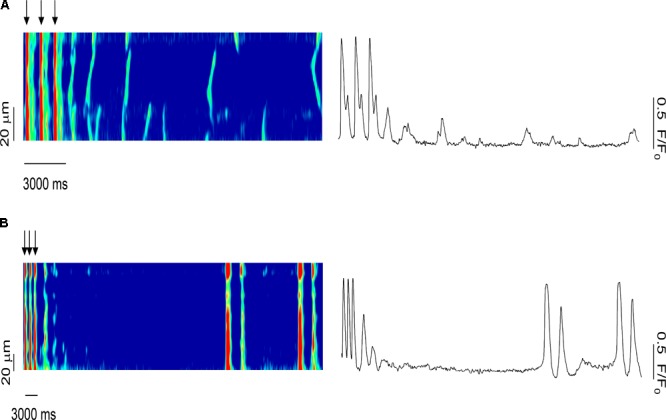
Role of systolic Ca^2+^ release in the synchronization of Ca^2+^ waves in the multicellular trabeculae (0.5 mM caffeine): effects of several pulses on the timing of the Ca^2+^ waves. **(A)** Last three stimulated Ca^2+^ transients before the stimulation stopped. A synchronized Ca^2+^ release event occurred immediately following the last stimulated Ca^2+^ release; however, over time the amplitude and temporal homogeneity of Ca^2+^ waves decreased. **(B)** A synchronized SR Ca^2+^ release occurred long after the last stimulated Ca^2+^ release in an area of the muscle that was previously completely quiescent, strongly suggesting of triggered activity elsewhere on the preparation that propagated via AP to this quiescent area. SC, stimulated contraction.

To investigate the role of systolic Ca^2+^ release in the synchronization of Ca^2+^ waves in a multicellular trabecula, we examined the effects of several stimulated pulses on the timing of diastolic Ca^2+^ release. **Figure 8A** shows the last three stimulated Ca^2+^ transients before the stimulation was stopped (0.5 mM Caffeine). Immediately following the last stimulated Ca^2+^ transient, there was a non-externally triggered Ca^2+^ release event that occurred uniformly throughout the myocytes; however, over time, this Ca^2+^ release decreased in both amplitude and temporal homogeneity among myocytes until there were only sporadic and spontaneous Ca^2+^ waves. Conversely, on occasion, there was a synchronized SR Ca^2+^ release event long after the stimulation had been removed (**Figure 8B**). Before this synchronized release, the myocytes in this line-scan were completely quiescent, but the amplitude of this Ca^2+^ transient was as large as in stimulated ones. This indicated that the Ca^2+^-transients in these myocytes were likely triggered by a propagating AP that originated elsewhere in this preparation.

## Discussion

The present study demonstrated that under conditions of Ca^2+^ dysregulation, i.e., in the presence of both isoproterenol and caffeine, diastolic SR Ca^2+^ release occurs with increasing synchronization among the myocytes *in situ* in isolated cardiac muscles in otherwise healthy rat myocardium. The Ca^2+^ dysregulation causes ESCs, and the amplitude of these ESCs ranged up to 100% of the amplitude of the developed force in electrically stimulated contractions. This latter finding suggests that all the myocytes were activated during some of these ESCs, which is in close agreement with the findings of our previous study regarding synchronization leading to non-externally stimulated APs in genetically manipulated murine myocardium (Brunello et al., 2013), and that synchronization depends on high SR calcium load (Aistrup et al., 2006; Wasserstrom et al., 2010). While we were not able to measure membrane potential simultaneously, the data suggest that many of these ESCs ultimately have the capability to stimulate extra-systolic APs. Using line-scan mode imaging in different locations within a muscle, we observed that the simultaneous Ca^2+^ release in a group of myocytes can initiate an non-externally triggered ESC, even well after repetitive electrical stimulation has stopped, and even in quiescent areas of the muscle. Taken together, the force measurements and the Ca^2+^ imaging experiments support the hypothesis that intracellular diastolic SR Ca^2+^ release can become synchronized in a multicellular cardiac preparation to the extent that it can involve many or all adjacent myocytes and evoke a triggered AP. Thus, this synchronization mechanism can occur in the absence of genetic mutations as shown by our previous study (Brunello et al., 2013) that was conducted in parallel to this study.

Under our experimental conditions of Ca^2+^ overload, that also cause human ventricular tissue to display ESCs (Elnakish et al., 2017), the relatively low dose of caffeine allows for increases in the open probability of the RyR2 without depleting the SR, and it decreases the threshold for SR Ca^2+^ release (Nieman and Eisner, 1985; Trafford et al., 2000). On the other hand, isoproterenol increases the SR Ca^2+^ content to a high enough level to reach the threshold for SR Ca^2+^ release (Venetucci et al., 2007). Therefore, when both a low concentration of caffeine and high concentrations of isoproterenol are present, diastolic Ca^2+^ waves occur in the majority of myocytes. This leaves less Ca^2+^ to be released during systole and effectively activate the myofilaments. The analysis of the amplitude of the force developed by the ESCs showed that a substantial number of ESCs had amplitudes >80% of the developed force in stimulated beats. We hypothesize that many of these high-amplitude ESCs would have the capability to stimulate extra-systolic APs and/or are the result thereof. The ESCs also displayed a high probability of occurrence around two or sometimes three specific time points. When three ESCs were present, we observed a clustering of ESCs around 309 ms after the stimulated contraction, with the second occurring at 577 ms and, if occurring, the third at 701 ms after the stimulated beat. The induction of a triggered arrhythmia requires not only spatial synchrony but temporal synchrony of Ca^2+^ release as well. We believe that the temporal synchronization is dictated by the previous stimulation that aligns the cellular RyR2 release. The time required for Ca^2+^ refilling in the SR and the RyR2 restitution determines the timing of the Ca^2+^ waves (Bers et al., 1993; Satoh et al., 1997).

To further examine the synchronization of Ca^2+^ release, a separate group of muscles were loaded with the Ca^2+^ indicator Rhod-2. In the control, non-Ca^2+^-overloaded muscles, we found simultaneous stimulated SR Ca^2+^ release in all six of the myocytes in the field of view, while in diastole no Ca^2+^ waves were observed. However, when both isoproterenol and caffeine were added Ca^2+^ waves were detected between the electrically stimulated Ca^2+^ release. Early after the addition of the drugs, not all of the myocytes demonstrated spontaneous Ca^2+^ release. After 5 min, however, the Ca^2+^ release typically occurred uniformly in each of the myocytes (four to seven) within the line-scan, indicating it was (non-externally) triggered rather than spontaneous. As the fluorescence profiles of the individual cells show, when the (spontaneous) Ca^2+^ release was asynchronous, the corresponding fluorescence signal peaked at different time points throughout diastole. However, when there was a synchronous Ca^2+^ release across all of the myocytes, the fluorescence peaked around the same time point in each cell. Consistent with the force experiments, this finding shows that with the addition of isoproterenol and caffeine diastolic Ca^2+^ release events do, indeed, occur, and these Ca^2+^ release events were able to synchronize across multiple myocytes in all the trabeculae examined. Gap junctions connect myocytes, and possibly calcium diffusion across these intercellular junctions can help contribute to a reduction in the threshold for a diastolic calcium release event. When a sufficient number of adjacent, coupled myocytes experience this reduction in threshold, it would increase the chance for the generation of a propagating AP.

When investigating the role of systolic Ca^2+^ release in the synchronization of Ca^2+^ waves, we observed that after the electrical stimulation had been stopped, a Ca^2+^ release event occurred uniformly throughout the myocytes. Over time, Ca^2+^ release decreased in both amplitude and temporal homogeneity until only sporadic Ca^2+^ waves occurred. Occasionally, a non-externally triggered whole-preparation synchronized SR Ca^2+^ release happened long after the stimulation had stopped. Since in the monitored area of the muscle there were no Ca^2+^ waves leading up to this uniform Ca^2+^ release, we conclude that it was the result of a triggering event that occurred from a distant cluster of myocytes and propagated to the field of view.

The vast majority of research in cardiomyocyte Ca^2+^ handling is primarily focused on individual myocytes; however, DADs and triggered arrhythmias in myocytes cannot directly be extrapolated to the intact heart, where the electrical coupling of myocytes acts as a “sink” for depolarizing currents (Joyner et al., 1983; Rohr et al., 1997; Spach and Boineau, 1997). On average, a ventricular myocyte is coupled with 11 other myocytes (Hoyt et al., 1989; Peters and Wit, 1998); when the membrane potential in a myocyte is altered by DADs, the depolarizing current will flow to the surrounding myocytes to minimize the voltage difference. DADs will be suppressed unless a sufficient number of neighboring myocytes synchronously develop a DAD. Several studies have used computer simulations and mathematical models to calculate the number of myocytes that are required for a triggered arrhythmia. It has been proposed that a DAD would have to arise in approximately 1000 myocytes for an ectopic beat to take place (Winslow et al., 1993), while other studies have calculated that a minimum of 700,000 to 800,000 myocytes are required for triggered arrhythmias to take place in the whole heart (Plotnikov et al., 2007; Xie et al., 2010). This number is derived from computer models that typically view the ventricular structure as a single connected entity. However, the endocardial surface of the mammalian heart has a highly trabeculated structure, with many hundreds of small linear muscle preparations, varying from 10 s of microns to a few millimeters in diameter, and from 1 mm to a few cm in length. In our experiments, the trabeculae investigated contained on average only 500–1000 myocytes. Within such a linear preparation, we observed that synchronized Ca^2+^ release was able to trigger a full-sized ESC in the entire preparation, including in parts of the muscle that were virtually completely quiescent, and were thus at least partially acting as an electrical sink. Anatomically, these trabeculae typically insert into the more solid ventricular mid-myocardium in wafer-like shapes, where the number of myocytes in the cross-section slowly increases as the trabecula inserts into the free wall. Many of the smaller muscles insert first into larger ones that, in turn, insert into the ventricular wall. We postulate that it is possible that this gradual change in the electrical sink capacity is not sufficient to quench the propagation of DADs, and may thus allow the trigger of an ESC. Future investigations will be needed to address this hypothesis.

## Conclusion

The present study demonstrates that diastolic SR Ca^2+^ release can become synchronized in multicellular trabeculae. Assessment of the developed force and intracellular Ca^2+^ suggests that the addition of both isoproterenol and caffeine can result in ESCs, and these events were able to spatially and temporally synchronize across multiple myocytes. Given the anatomy of the endocardial surface, it is feasible that triggered ventricular ESCs can be caused by a small, specifically localized group of myocytes, and may not necessarily require the much larger amounts of myocytes predicted by previously conducted modeling studies.

## Author Contributions

All authors listed have made a substantial, direct and intellectual contribution to the work, and approved it for publication.

## Conflict of Interest Statement

The authors declare that the research was conducted in the absence of any commercial or financial relationships that could be construed as a potential conflict of interest. The reviewer KL and handling Editor declared their shared affiliation.

## References

[B1] Aistrup G. L. Kelly J. E. Kapur S. Kowalczyk M. Sysman-Wolpin I. Kadish A. H. (2006). Pacing-induced heterogeneities in intracellular Ca^2+^ signaling, cardiac alternans, and ventricular arrhythmias in intact rat heart. *Circ. Res.* 99 e65–e73. 10.1161/01.RES.0000244087.36230.bf 16960102

[B2] Allen D. G. Kentish J. C. (1985). The cellular basis of the length-tension relation in cardiac muscle. *J. Mol. Cell. Cardiol.* 17 821–840. 10.1016/S0022-2828(85)80097-3 3900426

[B3] Bers D. M. Bassani R. A. Bassani J. W. Baudet S. Hryshko L. V. (1993). Paradoxical twitch potentiation after rest in cardiac muscle: increased fractional release of SR calcium. *J. Mol. Cell. Cardiol.* 25 1047–1057. 10.1006/jmcc.1993.1117 8283468

[B4] Bers D. (2001). *Excitation-Contraction Coupling and Cardiac Contractile Force* 2nd Edn. Dordrecht: Kluwer Academic Publishers 10.1007/978-94-010-0658-3

[B5] Biesiadecki B. J. Davis J. P. Ziolo M. T. Janssen P. M. L. (2014). Tri-modal regulation of cardiac muscle relaxation; intracellular calcium decline, thin filament deactivation, and cross-bridge cycling kinetics. *Biophys. Rev.* 6 273–289. 10.1007/s12551-014-0143-5 28510030 PMC4255972

[B6] Brunello L. Slabaugh J. L. Radwanski P. B. Ho H. T. Belevych A. E. Lou Q. (2013). Decreased RyR2 refractoriness determines myocardial synchronization of aberrant Ca^2+^ release in a genetic model of arrhythmia. *Proc. Natl. Acad. Sci. U.S.A.* 110 10312–10317. 10.1073/pnas.1300052110 23733959 PMC3690898

[B7] Elnakish M. T. Canan B. D. Kilic A. Mohler P. J. Janssen P. M. (2017). Effects of zacopride, a moderate IK1 channel agonist, on triggered arrhythmia and contractility in human ventricular myocardium. *Pharmacol. Res.* 115 309–318. 10.1016/j.phrs.2016.11.033 27914945 PMC5234043

[B8] Gyorke I. Hester N. Jones L. R. Gyorke S. (2004). The role of calsequestrin, triadin, and junctin in conferring cardiac ryanodine receptor responsiveness to luminal calcium. *Biophys. J.* 86 2121–2128. 10.1016/S0006-3495(04)74271-X 15041652 PMC1304063

[B9] Gyorke S. (2009). Molecular basis of catecholaminergic polymorphic ventricular tachycardia. *Heart Rhythm* 6 123–129. 10.1016/j.hrthm.2008.09.013 19121813

[B10] Gyorke S. Carnes C. (2008). Dysregulated sarcoplasmic reticulum calcium release: potential pharmacological target in cardiac disease. *Pharmacol. Ther.* 119 340–354. 10.1016/j.pharmthera.2008.06.002 18675300 PMC2798594

[B11] Gyorke S. Terentyev D. (2008). Modulation of ryanodine receptor by luminal calcium and accessory proteins in health and cardiac disease. *Cardiovasc. Res.* 77 245–255. 10.1093/cvr/cvm038 18006456

[B12] Hoyt R. H. Cohen M. L. Saffitz J. E. (1989). Distribution and three-dimensional structure of intercellular junctions in canine myocardium. *Circ. Res.* 64 563–574. 10.1161/01.RES.64.3.563 2645060

[B13] Janssen P. M. L. Stull L. B. Marban E. (2002). Myofilament properties comprise the rate-limiting step for cardiac relaxation at body temperature in the rat. *Am. J. Physiol. Heart Circ. Physiol.* 282 H499–H507. 10.1152/ajpheart.00595.2001 11788397

[B14] Joyner R. W. Picone J. Veenstra R. Rawling D. (1983). Propagation through electrically coupled cells. Effects of regional changes in membrane properties. *Circ. Res.* 53 526–534. 10.1161/01.RES.53.4.526 6627611

[B15] Kovacs M. Toth J. Hetenyi C. Malnasi-Csizmadia A. Sellers J. R. (2004). Mechanism of blebbistatin inhibition of myosin II. *J. Biol. Chem.* 279 35557–35563. 10.1074/jbc.M405319200 15205456

[B16] Lederer W. J. Tsien R. W. (1976). Transient inward current underlying arrhythmogenic effects of cardiotonic steroids in Purkinje fibres. *J. Physiol.* 263 73–100. 10.1113/jphysiol.1976.sp011622 1018270 PMC1307691

[B17] Monasky M. M. Biesiadecki B. J. Janssen P. M. (2010). Increased phosphorylation of tropomyosin, troponin I, and myosin light chain-2 after stretch in rabbit ventricular myocardium under physiological conditions. *J. Mol. Cell. Cardiol.* 48 1023–1028. 10.1016/j.yjmcc.2010.03.004 20298699 PMC2854324

[B18] Mulieri L. A. Hasenfuss G. Ittleman F. Blanchard E. M. Alpert N. R. (1989). Protection of human left ventricular myocardium from cutting injury with 2,3-butanedione monoxime. *Circ. Res.* 65 1441–1449. 10.1161/01.RES.65.5.1441 2805252

[B19] Nieman C. J. Eisner D. A. (1985). Effects of caffeine, tetracaine, and ryanodine on calcium-dependent oscillations in sheep cardiac Purkinje fibers. *J. Gen. Physiol.* 86 877–889. 10.1085/jgp.86.6.877 4078558 PMC2228797

[B20] Peters N. S. Wit A. L. (1998). Myocardial architecture and ventricular arrhythmogenesis. *Circulation* 97 1746–1754. 10.1161/01.CIR.97.17.1746 9591770

[B21] Plotnikov A. N. Shlapakova I. Szabolcs M. J. Danilo P. Jr. Lorell B. H. Potapova I. A. (2007). Xenografted adult human mesenchymal stem cells provide a platform for sustained biological pacemaker function in canine heart. *Circulation* 116 706–713. 10.1161/CIRCULATIONAHA.107.703231 17646577

[B22] Raman S. Kelley M. A. Janssen P. M. L. (2006). Effect of muscle dimensions on trabecular contractile performance under physiological conditions. *Pflugers Arch.* 451 625–630. 10.1007/s00424-005-1500-9 16082545

[B23] Rodriguez E. K. Hunter W. C. Royce M. J. Leppo M. K. Douglas A. S. Weisman H. F. (1992). A method to reconstruct myocardial sarcomere lengths and orientations at transmural sites in beating canine hearts. *Am. J. Physiol. Heart Circ. Physiol.* 263 H293–H306. 10.1152/ajpheart.1992.263.1.H293 1636767

[B24] Rohr S. Kucera J. P. Fast V. G. Kleber A. G. (1997). Paradoxical improvement of impulse conduction in cardiac tissue by partial cellular uncoupling. *Science* 275 841–844. 10.1126/science.275.5301.841 9012353

[B25] Satoh H. Blatter L. A. Bers D. M. (1997). Effects of [Ca2^+^]i, SR Ca2^+^ load, and rest on Ca2^+^ spark frequency in ventricular myocytes. *Am. J. Physiol.* 272 H657–H668. 10.1152/ajpheart.1997.272.2.H657 9124422

[B26] Schlotthauer K. Schattmann J. Bers D. M. Maier L. S. Schutt U. Minami K. (1998). Frequency-dependent changes in contribution of SR Ca2^+^ to Ca2^+^ transients in failing human myocardium assessed with ryanodine. *J. Mol. Cell. Cardiol.* 30 1285–1294. 10.1006/jmcc.1998.0690 9710797

[B27] Shannon T. R. Bers D. M. (1997). Assessment of intra-SR free [Ca] and buffering in rat heart. *Biophys. J.* 73 1524–1531. 10.1016/S0006-3495(97)78184-0 9284319 PMC1181051

[B28] Shannon T. R. Ginsburg K. S. Bers D. M. (2000). Potentiation of fractional sarcoplasmic reticulum calcium release by total and free intra-sarcoplasmic reticulum calcium concentration. *Biophys. J.* 78 334–343. 10.1016/S0006-3495(00)76596-9 10620297 PMC1300641

[B29] Shannon T. R. Ginsburg K. S. Bers D. M. (2002). Quantitative assessment of the SR Ca2^+^ leak-load relationship. *Circ. Res.* 91 594–600. 10.1161/01.RES.0000036914.12686.28 12364387

[B30] Slabaugh J. L. Brunello L. Gyorke S. Janssen P. M. (2012). Contractile parameters and occurrence of alternans in isolated rat myocardium at supra-physiological stimulation frequency. *Am. J. Physiol. Heart Circ. Physiol.* 302 H2267–H2275. 10.1152/ajpheart.01004.2011 22467303

[B31] Spach M. S. Boineau J. P. (1997). Microfibrosis produces electrical load variations due to loss of side-to-side cell connections: a major mechanism of structural heart disease arrhythmias. *Pacing Clin. Electrophysiol.* 20 397–413. 10.1111/j.1540-8159.1997.tb06199.x 9058844

[B32] Trafford A. W. Sibbring G. C. Diaz M. E. Eisner D. A. (2000). The effects of low concentrations of caffeine on spontaneous Ca release in isolated rat ventricular myocytes. *Cell Calcium* 28 269–276. 10.1054/ceca.2000.0156 11032782

[B33] Venetucci L. A. Trafford A. W. Diaz M. E. O’Neill S. C. Eisner D. A. (2006). Reducing ryanodine receptor open probability as a means to abolish spontaneous Ca^2+^ release and increase Ca^2+^ transient amplitude in adult ventricular myocytes. *Circ. Res.* 98 1299–1305. 10.1161/01.RES.0000222000.35500.65 16614307

[B34] Venetucci L. A. Trafford A. W. Eisner D. A. (2007). Increasing ryanodine receptor open probability alone does not produce arrhythmogenic calcium waves: threshold sarcoplasmic reticulum calcium content is required. *Circ. Res.* 100 105–111. 10.1161/01.RES.0000252828.17939.00 17110597

[B35] Wasserstrom J. A. Shiferaw Y. Chen W. Ramakrishna S. Patel H. Kelly J. E. (2010). Variability in timing of spontaneous calcium release in the intact rat heart is determined by the time course of sarcoplasmic reticulum calcium load. *Circ. Res.* 107 1117–1126. 10.1161/CIRCRESAHA.110.229294 20829511 PMC2967435

[B36] Winslow R. L. Varghese A. Noble D. Adlakha C. Hoythya A. (1993). Generation and propagation of ectopic beats induced by spatially localized Na-K pump inhibition in atrial network models. *Proc. Biol. Sci.* 254 55–61. 10.1098/rspb.1993.0126 8265676

[B37] Xie Y. Sato D. Garfinkel A. Qu Z. Weiss J. N. (2010). So little source, so much sink: requirements for afterdepolarizations to propagate in tissue. *Biophys. J.* 99 1408–1415. 10.1016/j.bpj.2010.06.042 20816052 PMC2931729

[B38] Zimmermann N. Boknik P. Gams E. Gsell S. Jones L. R. Maas R. (1996). Mechanisms of the contractile effects of 2,3-butanedione-monoxime in the mammalian heart. *Naunyn Schmiedebergs Arch. Pharmacol.* 354 431–436. 8897445 10.1007/BF00168433

